# mG-FAST, a single pre-hospital stroke screen for evaluating large vessel and non-large vessel strokes

**DOI:** 10.3389/fneur.2022.912119

**Published:** 2022-08-03

**Authors:** Roy El Koussa, Sarah Linder, Alicia Quayson, Shawn Banash, James J. MacNeal, Parshva Shah, Mariaelana Brenner, Ross Levine, Osama O. Zaidat, Vibhav Bansal

**Affiliations:** ^1^Department of Internal Medicine, Mercy Health Javon Bea Hospital, Rockford, IL, United States; ^2^Department of Interventional Neurology, Mercy Health Javon Bea Hospital, Rockford, IL, United States; ^3^Department of Emergency Medicine, Mercy Health Javon Bea Hospital, Rockford, IL, United States; ^4^College of Medicine, University of Illinois Rockford, Rockford, IL, United States; ^5^Department of Neurology, Mercy Health Hospital and Trauma Center, Janesville, WI, United States; ^6^Department of Neurology, Bon Secours Mercy Health St. Vincent Medical Center, Toledo, OH, United States

**Keywords:** stroke, stroke scale, stroke score, mG-FAST, G-FAST, pre-hospital screening, triage, large vessel occlusion

## Abstract

**Background:**

Several stroke scales have been implemented to enhance early recognition of large vessel occlusion (LVO) in the field. These scales necessitate a tiered approach requiring emergency medical services (EMS) to utilize two scales, one for identifying stroke and another for differentiating LVO from non-LVO. Ideally, a single stroke scale should be utilized by EMS for triage.

**Methods:**

This is a prospective analysis of 150 consecutive patients presenting with stroke symptoms from the field. The stroke scale modified Gaze-Face-Arm-Speech-Time (mG-FAST) was used to simultaneously identify stroke and detect LVO in the pre-hospital setting. Imaging was used to confirm the presence of a LVO and determine the sensitivity and specificity of mG-FAST. The receiver operating curve (ROC) was plotted to calculate the area under the curve (AUC). Youden's index was used to determine the optimal cutoff score. Inter-rater reliability was obtained by comparing the EMS and stroke provider mG-FAST scores. EMS dispatch-to-thrombectomy-capable stroke center (mothership, MS) arrival time and groin puncture time were compared before and after the implementation of mG-FAST.

**Results:**

33/150 patients had a confirmed LVO by imaging. 32/33 patients had an mG-FAST score ≥3. The AUC of mG-FAST was 0.899. An mG-FAST cut-off point of ≥3 yielded a sensitivity of 0.97 and specificity of 0.55 for LVO. The accuracy of this cut-off point was 64%. The EMS dispatch-to-MS time and groin puncture time decreased by 22 and 40 min after implementation of mG-FAST, respectively. With admission to the MS, the EMS dispatch-to-MS time decreased by 174.7 min compared to admission to a drip-and-ship (DS) hospital.

**Conclusions:**

Utilizing a single stroke scale in the field improves EMS dispatch-to-MS time, EMS dispatch-to-groin puncture time, and EMS door-to-intervention time. Implementation of mG-FAST as a pre-hospital screening tool is an effective method of triaging patients to the MS or DS hospitals.

## Introduction

There are ~800,000 new ischemic strokes in the United States annually (1). Of these, close to 20% are the result of a large vessel occlusion (LVO) (2). A large vessel occlusion is defined by a thrombus in one of the major cerebral vessels including the terminal internal carotid artery (ICA), middle cerebral artery (MCA), anterior cerebral artery (ACA), or basilar arteries. Studies have shown that pharmacologic thrombolytic therapy alone for LVOs is often ineffective for achieving artery recanalization. One study suggested a success rate of <1% with IV thrombolytic alone (3).

Mechanical thrombectomy (MT) has become the international standard of care for the treatment of patients who are suffering from an acute LVO. A recent meta-analysis comparing different treatments of LVO showed that MT combined with intravenous thrombolysis is the safest and most effective treatment modality with a successful recanalization rate of 75% (4). Early revascularization improves outcomes of patients with LVO with research demonstrating an average of 90 min saved if patients are admitted directly to a MS hospital (5). It is therefore imperative that patients who are suffering from an LVO are transferred to MS hospitals without delay. To accomplish this, a simple and effective screening tool for EMS to use in the field is necessary to simultaneously identify stroke patients and LVO. This chosen tool should have a high sensitivity and negative predictive value to ensure that no LVO is missed, while maintaining a low false positive rate to avoid overwhelming endovascular capable centers.

Several LVO stroke scales have been implemented in communities to enhance early recognition of LVO. However, these scales necessitate a tiered approach requiring EMS to either utilize two separate stroke scales, or complete complicated physical exams, unfamiliar to EMS and difficult to perform in the field. In the emergent setting, it is inefficient for multiple scales to be used for a single disease process.

Ideally, a single stroke scale should be utilized by EMS to triage all strokes, including LVO. The scale should be validated in the pre-hospital setting in a suspected stroke cohort, inclusive of stroke mimics. EMS professionals around the country are familiar with the Cincinnati Stroke Scale (Face-Arm-Speech-Time, FAST) which has high sensitivity for stroke. A score to identify LVO, that builds on this well-known scale, would be readily utilized by EMS to simultaneously screen for stroke and LVO. The G-FAST scale incorporates gaze deviation in FAST to preserve the high sensitivity of FAST for stroke detection with the added benefit of allowing simultaneous evaluation for LVO. The presence of gaze deviation is the single best predictor of LVO on the National Institute of Health Stroke Scale (NIHSS). It has a sensitivity and specificity for LVO of 58 and 95%, respectively (6).

Prior retrospective studies have validated the accuracy of G-FAST, with a score of 3 as predictive of LVO and comparable to that of NIHSS (7). Our study proposes a modified version of G-FAST, termed mG-FAST. mG-FAST differs from G-FAST in giving gaze two points, therefore placing higher weight on a cortical sign indicative of LVO. We propose the modified mG-FAST scale be used for simultaneously triaging of stroke and LVO. In this study, we aim to validate the mG-FAST score as a screening tool for stroke and LVO, and to determine if its implementation improves treatment times for stroke intervention.

## Materials and methods

### Setting

This is a prospective study that took place in a region with an estimated population of 700,000. The region has 12 community hospitals representing both rural and suburban settings. All three levels of stroke care are represented within this region which includes: one hospital without a state Department of Public Health stroke designation, seven Acute Stroke Ready hospitals, three Primary Stroke Center hospitals, and two Comprehensive Stroke Center hospitals. Of these hospitals, the stroke team for this study operates at two facilities: one primary stroke center and one comprehensive stroke center. This study reports the findings and outcomes of patients presenting at the comprehensive stroke center.

### Rationale for mG-FAST

The modified Gaze-Face-Arm-Speech-Time builds on the validated and widely used Cincinnati stroke scale (FAST) and G-FAST to enhance their ability to differentiate LVO from non-LVO strokes. Similarly to G-FAST, it uses the same variables: gaze deviation, facial asymmetry, motor weakness, and aphasia. However, gaze deviation is attributed double the weight of the other variables included in the scale (Table 1).

**Table 1:** mG-FAST variables and their attributed weights.

**Variable**	**Score**	**Definition**
Gaze deviation	0–2	0: Normal
		2: Partial or total gaze paresis
Facial palsy	0–1	0: Normal
		1: Partial or total paralysis
Arm weakness	0–1	0: No drift or mild drift
		1: Moderate drift or paralysis
Speech	0–1	0: Normal
		1: Aphasia

Cortical signs and symptoms such as gaze deviation, neglect, and aphasia are better predictors of LVO strokes than motor symptoms. Moreover, EMS personnel are familiar with evaluation of gaze deviation since it is a part of G-FAST, the scale used prior to the implementation of mG-FAST. Since our scale was designed for pre-hospital triage, it was of utmost importance to choose variables that are easily and reliably assessed by EMS personnel, hence gaze deviation was the cortical sign of choice.

### EMS training

EMS leadership formed a local hospital stroke care committee to design and monitor the educational content of a stroke training module. Spanning over four individual sessions, a stroke neurologist provided comprehensive training and material, using the approved stroke training module, to EMS personnel. The training material included a 2-h lecture on the mG-FAST exam as well as stroke mimics, acute stroke syndromes, and pre-hospital management of strokes. In addition, EMS educators performed two individual training sessions in EMS facilities that participated in training of EMS personnel.

In October 2018, the state offices of EMS and Department of Public Health (DPH) approved the use of mG-FAST in suspected stroke patients. In November 2018, the region adopted the use of the mG-FAST as the stroke assessment tool for EMS providers. EMS personnel were instructed to provide a mG-FAST score for any suspected stroke patients with last known normal (LKN) <24 h prior to presentation, wake-up stroke, or with an unknown LKN.

### Pre-hospital protocols

EMS protocol was established for patients presenting with stroke symptoms and an mG-FAST ≥1. An mG-FAST of 1 or more followed previous FAST guidelines, activating a code stroke response and routing to the nearest mothership (MS) or drip-and-ship (DS) hospitals. If the mG-FAST is ≥3, and the time to the nearest MS is >15 min, the EMS would take the patient to the nearest DS hospital. If the time to the nearest MS is <15 min, the EMS would take the patient to that MS hospital. The process included pre-arrival notification by EMS personnel with the time of the LKN, estimated time of arrival to the emergency department (ED), age, gender, and mG-FAST score. The notification was sent out as a stroke alert page to the acute stroke team. The latter consisted of a stroke neurologist, neuro-interventionalist, neurology nurse practitioner, stroke coordinator, bed flow coordinator, computed tomography (CT) scan staff, intensive care unit (ICU) charge nurse and phlebotomist. Patients were registered immediately upon arrival to the hospital. After a brief assessment by the ED physician, patients were rapidly escorted to the CT scan for a CT head, CT angiogram head and neck, and CT perfusion (if LKN was >6 h or unknown). A member of the stroke team performed the mG-FAST scale and NIHSS on presentation.

### Data collection

Institutional review board approval was obtained to maintain a registry of stroke alert patients and to perform the research study. Data in the registry included: demographics, risk factors, EMS and stroke provider mG-FAST score, diagnostic imaging findings, treatments/procedures performed, treatment times, and modified Rankin Scale at 3-month follow up. Patients were excluded if a stroke alert was not called ahead by EMS personnel or if the patient was <18 years old. This study also did not include patients who had the initial stroke alert called in the ED or during hospitalization to maintain the integrity of validating the tool for EMS use.

### Statistical analysis

Statistical analysis was performed using descriptive statistics *via* Statistical Analysis Software (SAS). The aim of the analysis was to validate the scale as an LVO assessment tool for EMS use and determine its sensitivity and specificity. One hundred fifty consecutive patients were rated with this scale by EMS professionals in the pre-hospital setting and independently evaluated by a member of the stroke team upon arrival to the hospital. The receiver operating curve (ROC) was used to calculate the area under the curve (AUC). Youden's index was calculated to determine the optimal cutoff point. The degree of inter-rater agreement was determined by calculation of the Cohen's κ statistic and percent agreement calculation using test-retest methodology. EMS dispatch-to-MS arrival time, EMS dispatch-to-arterial puncture time, door-to-arterial puncture time and door-to-intervention time were measured for the 15 months prior to and 18 months after implementation of mG-FAST.

## Results

### Demographics

Of the 150 consecutive patients included in the study, 82 patients were males and 68 patients were females. The age range was between 28 and 100 years. The mean age for males was 68.18 (SD ± 13.05). The mean age for females was 72.78 (SD ± 1 5.98). Forty percent of patients were smokers. The baseline characteristics of included patients are summarized in Table 2.

**Table 2:** Baseline characteristics of included patients.

**Baseline characteristic**	**Number of patients**	**Mean/Frequency (%)**
**Age (years)**	150	70.3 (±14.5)
**Sex (%)**		
Male	82	54.6%
Female	68	45.4%
**Comorbidities (%)**		
**Atrial fibrillation**	26	17%
Hypertension	123	82%
Hyperlipidemia	88	58.7%
Type II DM	118	79%
CAD/CHF	48	32%
History of stroke	38	25.3%
CKD 3B or worse	12	8%
Known clotting disorder	2	1%
Active cancer	9	6%
**Stroke etiology (%)**		
Large vessel	33	22%
Small vessel	23	15.4%
Mimic	53	35.3%
Hemorrhage	15	10%
Undetermined	26	17.3%
**Treatment (%)**		
Thrombectomy	33	22%
IV Thrombolysis	6^¶^	4%
Stent/coil	25	16.67%
Supportive care	87	58%

*CAD, coronary artery disease; CHF, congestive heart failure; CKD, chronic kidney disease; DM, diabetes mellitus*.

¶ *One patient received both IV thrombolysis and mechanical thrombectomy*.

### Ischemic stroke mG-FAST

Of the 150 stroke alerts with mG-FAST called by EMS in the field and admitted through the emergency department, 33 patients had vascular imaging confirming a LVO in the anterior cerebral artery A1 segment (ACA), middle cerebral artery (MCA) M1 or M2 segment, internal carotid artery (ICA) terminus, or basilar artery (Table 3). Out of those, 32 (97%) patients had an mG-FAST score of 3 or more and an NIHSS >6 (mean NIHSS of 14). One patient (3%) with a LVO had an mG-FAST score <3 and NIHSS <6 (Table 4). The majority of occlusions were MCA occlusions.

**Table 3:** Distribution of vessel occlusions in the study population.

**Artery**	**Number of patients**	**Frequency (%)**
MCA	18	55%
ACA	8	24%
ICA	5	15%
Basilar	2	6%

*MCA, middle cerebral artery; ACA, anterior cerebral artery; ICA, internal carotid artery*.

**Table 4:** Distribution of EMS mG-FAST scores of LVO and non-LVO stroke patients.

**mG-FAST**	**LVO**	**Non-LVO**	**Total**
1	0	30	30
2	1	34	35
3	11	30	41
4	7	11	18
5	14	12	26
Total	33	117	150

### Inter-rater reliability

mG-FAST had an inter-rater reliability between EMS and stroke providers of 83% matched (*n* = 124/150) and 17% unmatched (*n* = 26/150). Cohen's kappa statistic was 0.65 (*p* < 0.0001). The 26 patients with unmatched stroke scale score between EMS and stroke providers were patients with symptoms that resolved prior to arrival to the ED. Of the 33 LVO patients, there was 100% congruency of the mG-FAST score between EMS and stroke providers.

### Predictive value of mG-FAST for LVO

**Figure 1:**
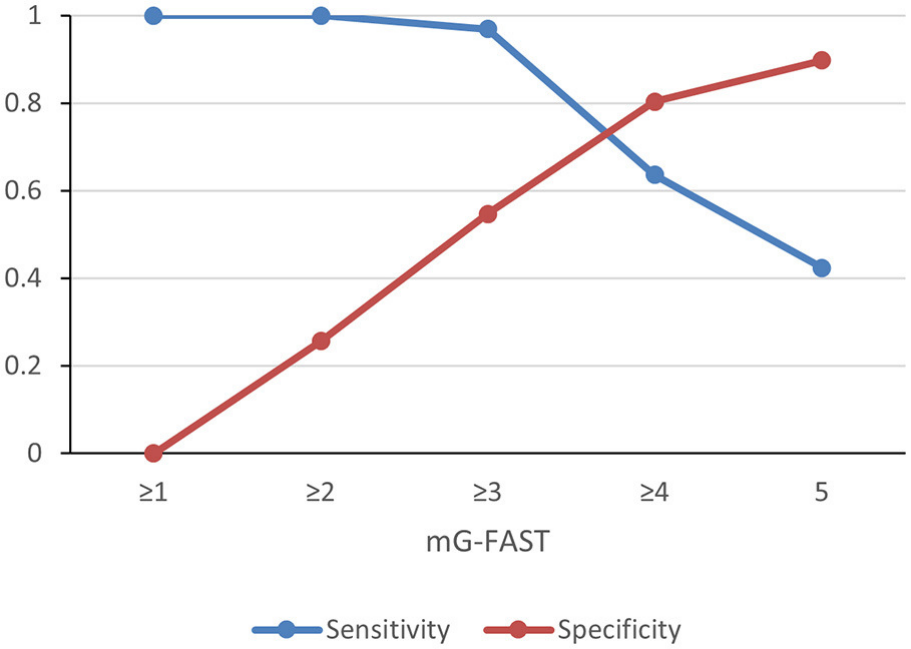
Sensitivity and specificity of different cutoff points of mG-FAST for detection of large vessel occlusion strokes.

The AUC for mG-FAST was 0.899. The highest Youden's index was achieved for mG-FAST ≥3. An mG-FAST cut-off point of ≥3 by EMS yielded a sensitivity of 0.97 and specificity of 0.55 for LVO (Figure 1). Negative predictive value (NPV) was 0.98 and positive predictive value (PPV) of 0.38. The accuracy of this cut-off point was 64% (CI 95%: 55.77–71.67%).

### Comparison to other LVO scales

An mG-FAST score of 3 was found to have a higher sensitivity and NPV when compared to all other EMS LVO scales (Table 5) (7, 8).

**Table 5:** Statistical parameters of most commonly used stroke scales.

**Tool/cutoff**	***n*** **/*N***	**Sensitivity**	**Specificity**	**NPV**	**PPV**	**Accuracy**
mG-FAST ≥ 3	33/150	97	55	98	38	64
NIHSS ≥ 12	1,420/3,505	69.5	69.8	45.0	86.5	69.7
FAST = 3	2,207/3,505	81.2	43.5	33.8	86.7	53.4
G-FAST = 4	1,029/3,505	54.3	79.5	48.5	83.0	53.4
C-STAT ≥ 2	1,461/3,505	71.1	68.8	44.8	87.0	69.4
3-ISS ≥ 2	1,283/3,505	64.6	73.4	46.3	85.3	71.1
PASS ≥ 2	1,802/3,505	77.3	59.3	40.3	88.0	64.0
RACE ≥ 5	1,442/3,505	68.9	68.8	44.0	86.1	68.9
VAN	19/62	100	90	74	100	NA

*3-ISS, 3 item stroke scale; C-STAT, Cincinnati stroke triage assessment tool; FAST, field assessment stroke triage; G-FAST, gaze, face, arm, speech, time test; mG-FAST, modified gaze, face, arm, speech, time test; NIHSS, national institutes of health stroke scale; PASS, postural assessment scale for stroke; RACE, rapid arterial occlusion evaluation; VAN, vision, aphasia, neglect*.

### Non-LVO strokes with mG-FAST ≥3

Of the 150 patients called as code stroke by EMS, 117 patients were without LVO. Of these patients, 53 (45.3%) had an mG-FAST score of 3, 4, or 5 and 64 (54.7%) had an mG-FAST score of 1 or 2. Of the 53 patients with an mG-FAST score of 3, 4, or 5, 19 (35.8%) were mimics, 12 (22.6%) were small vessel or distal MCA infarcts, 10 (18.9%) had carotid stenosis requiring revascularization, 2 (3.8%) were aneurysmal subarachnoid hemorrhage (SAH) requiring embolization, and 11 (21%) were intra-cerebral hemorrhages. Seizure was the most common stroke mimic.

### Improving response time

Following implementation of mG-FAST, mean EMS dispatch-to-MS time decreased by 22 min; mean EMS dispatch-to-arterial puncture time decreased by 62 min; mean door-to-groin puncture time decreased by 27 min. Mean door-to-intervention time also decreased by 50 min.

Out of the 32 LVO patients with an mG-FAST of 3 or more, 10 were admitted to the MS hospital and 12 were admitted to a DS hospital. The mean EMS dispatch-to-MS time was 53.9 and 228.6 min, respectively. This resulted in a decrease of 174.7 min.

## Discussion

mG-FAST differs from G-FAST by giving greater weight (2 points) to gaze preference, the best predictor of LVO. This study prospectively enrolled 150 consecutive patients identified by EMS as potential strokes using only the mG-FAST scale. Patients with a score of 1 or 2 were paged as a non-LVO code stroke and those with an mG-FAST ≥3 as an LVO code stroke.

An mG-FAST score of 3 was chosen as the threshold for LVO for two main reasons. First, the presence of gaze preference (2 points) and hemiparesis of an extremity (1 point) are very predictive of LVO, therefore yielding a score of 3. Secondly, prior studies have demonstrated that the presence of all 3 signs of the FAST scale, which would also result in an mG-FAST score of 3, as highly predictive of LVO even in the absence of gaze deviation (9).

mG-FAST has a very high sensitivity and NPV making it a well-suited LVO screening tool. No posterior circulation strokes were missed from the field using mG-FAST. The scale allowed EMS to identify all but one LVO in the field. This one patient, who had a mG-FAST score of 2, had an LVO due to intracranial atherosclerotic disease (ICAD), an etiology that does not bode well with thrombectomy. This false negative rate of 3% is consistent with current recommendations that aim for an under-diagnosis rate of ≤10%; it is the lowest of all validated scales (10).

Compared to most other LVO scales, mG-FAST has one of the highest sensitivities and NPV. Although VAN resulted in similar sensitivity and NPV, its real-world application is limited as it was validated amongst emergency room registered nurses and physicians rather than EMS personnel, where the scale has its greatest applicability. This tool is validated amongst a variety of EMS training and experience levels, both in the rural and urban settings. Moreover, tools which use neglect, a component of VAN, has only fair inter-rater reliability (11). The inter-rater reliability of mG-FAST was 83%, further supporting the use of mG-FAST as a screening test in the field to better triage suspected strokes to appropriate medical centers.

PRESTO, a recently published study comparing multiple available stroke scales demonstrated that RACE, G-FAST, and CG-FAST approached the performance of NIHSS in detecting LVO (12). Similarly to our suggested scale, all three of these scales include cortical signs, highly specific for LVO. However, mG-FAST is easier to perform in the field than RACE and CG-FAST, and it attributes heavier weight to gaze deviation than G-FAST. Owing to this, mG-FAST will be easier to adopt without compromising performance as a screening tool.

Pre-hospital stroke triage tools with high false positive rates have the risk of overburdening comprehensive stroke centers. An acceptable rate of overdiagnosis is 30–50% (10). mG-FAST has a false positive rate of 45% which is within this range. Moreover, this false positive rate includes patients who would benefit from a center with high level comprehensive neurologic care including brain tumor, status epilepticus and cerebral hemorrhage.

Of note, posterior circulation strokes were included in our study despite the difference in clinical presentation compared to anterior circulation strokes. Signs and symptoms of posterior circulation strokes include vertigo, nausea/vomiting, visual field defects, limb weakness, gait ataxia, and dysarthria, among others. Majority of these symptoms are not included in pre-hospital stroke scales, including mG-FAST. That being said, the goal of any pre-hospital stroke scale is to identify any stroke in the field, including posterior circulation strokes. Although anterior and posterior circulation strokes do not share the same presentation, there is some overlap in signs and symptoms. We feel that including posterior circulation strokes in our analysis is important to adequately represent real-world application of our suggested scale.

EMS had 100% compliance in utilizing the mG-FAST scale. This compliance was likely related to EMS personnel's familiarity with many of the features of mG-FAST, as the scale adds a single additional sign, gaze preference, to the universally known Cincinnati stroke scale (FAST). The successful implementation of mG-FAST in our diverse EMS community, which included rural and volunteer personnel, underscores its broad applicability in other communities.

Our study demonstrates that EMS providers can effectively utilize mG-FAST as a single scale for evaluating stroke and large vessel occlusion (LVO). The use of mG-FAST drastically decreased the EMS dispatch-to-MS time, EMS dispatch-to-groin puncture time, and the EMS door-to-intervention time. Additionally, this study demonstrated that using mG-FAST as a pre-hospital LVO screening tool was an effective and efficient method in triaging patients to a MS hospital.

Our study is not without limitations. First, we did not prospectively compare mG-FAST to other LVO screens. Second, the total sample size, as well as the sample size of LVO involving the posterior circulation were small. This limited the power of our study and further validation studies in larger cohorts are needed to confirm our findings. Third, only patients that presented to one out of nine hospitals in the geographic study area were included in the present study. It's important to note though that the hospital is actually one of only two thrombectomy-capable centers in the area. Finally, the enhanced sensitivity of mG-FAST resulted in lower specificity and the inclusion of more ischemic stroke mimics such as status epilepticus. However, as noted above, these conditions also have improved outcomes when treated at comprehensive stroke centers.

Utilizing a single comprehensive stroke assessment tool in the field improved EMS dispatch-to-MS time as well as EMS dispatch-to-groin puncture time in patients with LVO stroke. This study demonstrated that using mG-FAST as a pre-hospital stroke screening tool was an effective and efficient method in triaging patients into MS and DS hospitals. mG-FAST is the only scale to our knowledge that has been prospectively validated in the pre-hospital setting in a suspected stroke cohort that allows the use of a single stroke scale to triage all strokes, including LVO. This scale has the highest sensitivity and highest negative predictive value of all stroke scales prospectively studied in the field. The utilization of this single scale for EMS to triage all stroke patients results in a simple, reliable, and effective tool ensuring a high level of compliance.

## Data availability statement

The raw data supporting the conclusions of this article will be made available by the authors, without undue reservation.

## Ethics statement

The studies involving human participants were reviewed and approved by Mercy Health Corporation IRB. Written informed consent for participation was not required for this study in accordance with the national legislation and the institutional requirements.

## Author contributions

REK, SL, VB, SB, and MB drafted and revised the manuscript. REK and PS conducted the statistical analyses. VB, SL, AQ, SB, and JM participated in the design of the study. JM and VB conducted the education of EMS personnel. All authors made contributions in revising, editing, and proofreading the final version of the manuscript.

## Conflict of interest

The authors declare that the research was conducted in the absence of any commercial or financial relationships that could be construed as a potential conflict of interest.

## Publisher's note

All claims expressed in this article are solely those of the authors and do not necessarily represent those of their affiliated organizations, or those of the publisher, the editors and the reviewers. Any product that may be evaluated in this article, or claim that may be made by its manufacturer, is not guaranteed or endorsed by the publisher.

## References

[1] Writing Group Members Mozaffarian D Benjamin EJ Go AS Arnett DK Blaha MJ. Executive summary: heart disease and stroke statistics−2016 update: a report from the American Heart Association. Circulation. (2016) 133:447–54. 10.1161/CIR.0000000000000366 26811276

[2] Waqas M Mokin M Primiani CT Gong AD Rai HH Chin F. Large vessel occlusion in acute ischemic stroke patients: a dual-center estimate based on a broad definition of occlusion site. J Stroke Cerebrovasc Dis. (2020) 29:104504. 10.1016/j.jstrokecerebrovasdis.2019.104504 31761735

[3] Tsivgoulis G Katsanos AH Schellinger PD Ktroke patients: a dual-center es. Successful reperfusion with intravenous thrombolysis preceding mechanical thrombectomy in large-vessel occlusions. Stroke. (2018) 49:232–5. 10.1161/STROKEAHA.117.019261 29212743 PMC5742056

[4] Hui W Wu C Zhao W Sun H Hao J Liang H. Efficacy and safety of recanalization therapy for acute ischemic stroke with large vessel occlusion. Stroke. (2020) 51:2026–35. 10.1161/STROKEAHA.119.028624 32486966

[5] Mueller-Kronast N Froehler MT Jahan R Zaidat O Liebeskind D Saver JL. Impact of EMS bypass to endovascular capable hospitals: geospatial modeling analysis of the US STRATIS registry. J Neurointerv Surg. (2020) 12:1058–63. 10.1136/neurintsurg-2019-015593 32385089 PMC7569363

[6] Taqi MA Sodhi A Suriya SS Quadri SA Farooqui M Salvucci AA. Design, application and infield validation of a pre-hospital emergent large vessel occlusion screening tool: ventura emergent large vessel occlusion score. J Stroke Cerebrovasc Dis. (2019) 28:728–34. 10.1016/j.jstrokecerebrovasdis.2018.11.014 30591260

[7] Scheitz JF Abdul-Rahim AH MacIsaac RL Cooray C Sucharew H Kleindorfer D. Clinical selection strategies to identify ischemic stroke patients with large anterior vessel occlusion: results from SITS-ISTR (safe implementation of thrombolysis in stroke international stroke thrombolysis registry). Stroke. (2017) 48:290–7. 10.1161/STROKEAHA.116.014431 28087804

[8] Teleb MS Ver Hage A Carter J Jayaraman MV McTaggart RA. Stroke vision, aphasia, neglect (VAN) assessment-a novel emergent large vessel occlusion screening tool: pilot study and comparison with current clinical severity indices. J Neurointerv Surg. (2017) 9:122–6. 10.1136/neurintsurg-2015-012131 26891627 PMC5284468

[9] Zachrison KS Khatri P. Self-driven prehospital triage decisions for suspected stroke-another step closer. JAMA Neurol. (2021) 78:146–8. 10.1001/jamaneurol.2020.4425 33252636

[10] Michel P. Prehospital scales for large vessel occlusion: closing in on a moving target. Stroke. (2017) 48:247–9. 10.1161/STROKEAHA.116.015511 28087805

[11] Keenan KJ Kircher C McMullan JT. Prehospital prediction of large vessel occlusion in suspected stroke patients. Curr Atheroscler Rep. (2018) 247–9. 10.1007/s11883-018-0734-x 29781051

[12] Duvekot MHC Venema E Rozeman AD Moudrous W Vermeij FH Biekart M. Comparison of eight prehospital stroke scales to detect intracranial large-vessel occlusion in suspected stroke (PRESTO): a prospective observational study. Lancet Neurol. (2021) 20:213–21. 10.1016/S1474-4422(20)30439-7 33422191

